# Cognition and reproductive success in cowbirds

**DOI:** 10.3758/s13420-021-00506-0

**Published:** 2021-12-16

**Authors:** David J. White, J. Arthur, H. B. Davies, M. F. Guigueno

**Affiliations:** 1 Department of Psychology, Wilfrid Laurier University, 75 University Ave. W, Waterloo, ON N2L 3C5 Canada; 2 Laboratory of Animal Medicine, Stanford University School of Medicine, Stanford, CA USA; 3 Department of Biology, McGill University, Montreal, Quebec Canada

**Keywords:** Reproductive success, Cognition, Spatial memory, Color memory, Sex differences, Brown-headed cowbird, Birdsong, Mate choice

## Abstract

Understanding the relationships between cognitive abilities and fitness is integral to an evolutionary study of brain and behavior. However, these relationships are often difficult to measure and detect. Here we draw upon an opportunistic sample of brown-headed cowbird (*Molothrus ater*) subjects that had two separate research experiences: First, they engaged in a large series of cognitive tests in David Sherry’s Lab in the Advanced Facility for Avian Research (AFAR) at Western University, then subsequently moved to the Field Avian Research Megalab (FARM) at Wilfrid Laurier University where they lived in large breeding flocks in aviaries with other wild-caught cowbirds. Thus, we had extensive measures of cognitive abilities, breeding behavior, and reproductive success for these birds. We report here, for the fist time, the surprisingly strong connections we found among these different measures. Female cowbirds’ spatial cognitive abilities correlated positively with how intensely they were courted by males, and with their overall egg production. Males’ spatial cognition correlated positively with their ability to engage in singing contests (“countersinging”) with other males. In addition, a separate non-spatial cognitive ability correlated positively with the attractiveness of the songs they sung. In sum, these results suggest the cognitive skills assessed in the lab were strongly connected to breeding behavior and reproductive success. Moreover, since certain cognitive abilities related to different aspects of breeding success, it suggests that cognitive modules may have specialized adaptive value, but also that these specialized skills may interact and influence fitness in surprising ways.

## Introduction

The integration of evolutionary theory into studies of the brain and its processes created a paradigmatic shift in the study of learning and behavior (Rozin & Kalat, 1971; Shettleworth, 2010). Sherry and colleagues (Sherry, 1982; Sherry, 2006; Sherry et al., 1993; Sherry & Schacter, 1987) conducted some of the seminal theoretical and experimental work in this area. Sherry (2006) used the term “Neuroecology” to describe the approach of integrating neuroscience, comparative psychology, and behavioral ecology to examine the form and function of memory systems. This approach provided a new testable framework for understanding information processing in animals, where discrete cognitive modules and the neural systems underpinning them could be considered adaptations, shaped and organized by the principles of natural and sexual selection, to have evolved to overcome the ecological demands imposed on the animal (Sherry & Schacter, 1987). This approach has had spectacular success in providing new insights into how the brain is organized and functions.

One challenge involved in taking an integrative approach to studying cognition is that it requires examining both mechanism and function, which is often beyond the scope of one research program. Mechanistic approaches that study the neurobiology and stimulus control of cognitive processes typically require the laboratory, where confounding variables like prior experience, motivation, and social learning can be controlled. The laboratory, however, often does not allow for measurement of the fitness consequences of cognitive abilities. These types of questions most often must be studied in the wild. As a consequence, collecting the full complement of data for any given study system remains a distinct challenge (Boogert et al., 2018). The literature that documents the link between variation in cognitive performance and fitness is small. Recently however, several research groups have made progress measuring cognition in the wild and linking it to fitness. In most cases of these cases, fitness is measured by survival (Ashton et al., 2018; Benedict et al., 2020; Huebner et al., 2018; Madden et al., 2018; Maille & Schradin, 2016; Sonnenberg et al., 2019).

The literature documenting effects of cognition on fitness when fitness is measured by reproductive success is even sparser (for examples, see: Branch et al., 2019; Chen et al., 2019; Medina-Garcia & Wright, 2021; Preiszner et al., 2017; Shaw et al., 2019). Successful reproduction is a product of a lifetime of interactions with the world, and thus documenting the effect of any one aspect of cognition on reproductive success can be less than straightforward. For example, the efficacy of cognitive abilities may vary by context, being useful in some environments but not others (Cole et al., 2012), cognitive skills may interact positively or negatively with each other (Kawecki, 2003), and alternative cognitive strategies for dealing with specific challenges may exist (White et al., 2017).

Cole et al. (2012) provide an illuminating example of a complex interaction between cognition and reproductive success. In great tits, *Parus major,* highly accurate problem solvers produced larger clutches than poor problem solvers, but highly accurate birds were also more likely to be so hypervigilant for predators that they deserted nests more often, costing them some degree of reproductive success (see also Johnson-Ulrich et al., 2019; Wetzel, 2017).

Our attempt to deal with many of these challenges and connect cognition and reproductive success focuses on one of the original model systems of neuroecology that began with Sherry et al. (1993): the brown-headed cowbird.

Brown-headed cowbirds are common North American songbirds. Their breeding system, however, is different than most. Cowbirds are obligate brood parasites and thus females have the distinct challenge each breeding season to prospect for the nests of host species that will serve as foster parents for their young (Friedmann, 1929). This breeding strategy presents females with a problem to solve: they must find suitable nests for their eggs and remember those locations when it comes time to lay. It stands to reason that those females with better spatial memory abilities who can find and remember the locations of more nests would have more breeding opportunities and therefore gain higher levels of reproductive success. Sherry et al. (1993) provided substantial neurobiological support for this idea. They showed that in female cowbirds the relative volume of the hippocampus – the area of the brain associated with spatial memory ability – was larger than other closely related, non-brood parasitic species and larger than males of their species. Similar patterns have been found in other species of brood parasitic cowbirds (shiny cowbirds *Molothrus bonariensis*; Clayton et al., 1997; Reboreda et al., 1996). Furthermore, female brown-headed cowbirds demonstrate enhanced neurogenesis in hippocampus leading up to the breeding season when spatial memory demands are highest (Guigieno et al., 2016).

In the lab, Guigueno et al. (2014) have studied the performance of female and male cowbirds across several types of cognitive tasks. They have found that females excelled over males at spatial tasks involving finding a location paired with reward. In other types of tasks, however – color discrimination, for example males and females do not differ. Also, depending on the type of task – touchscreen delayed match-to-sample tests versus open-field discovery, for example – males can perform as well, if not better than, females (Guigueno et al., 2015). Finally, performance by both males and females can improve depending on the time of the year (when birds are either in or out of breeding condition; Guigueno et al., 2014). Thus, the behavioral results from the lab are complicated (see also similar work in shiny cowbirds where males actually outperform females in spatial tasks; Astie et al., 1998), but do suggest, at least for brown-headed cowbirds, superiority for females in spatial tasks, and there seem to exist different types of cognitive abilities that influence performance in different types of tasks.

White and colleagues have studied the cognitive processes that female cowbirds use when prospecting for nests in aviaries (Davies & White, 2018; White, 2019; White et al., 2007a, b, 2009; White et al., 2017). This work has revealed that females are extremely adept at finding nests, and once found, select among nests based on size, pattern, and the number of eggs present. White et al. (2017) also found that females vary in their ability to select viable nests. The less-skilled individuals, however, were able to compensate with another cognitive skill: they followed other females to high-quality nests, copied their nest selection, removed the prospecting female’s egg, and laid their own. In sum, while work on the connection between cognition and effective parasitism is limited, it does suggest that spatial cognition is one important variable associated with effective parasitism, but not the only one.

### Cognition’s contribution to fitness

Patterns of egg laying in the wild are extremely difficult to track, given that females range over a large area when prospecting and they parasitize a large assortment of different species (Friedmann, 1963; but see Louder et al., 2019; Woolfenden et al., 2003). Work from aviaries has revealed there are many variables that have critical links to reproductive success, which also have the potential to complicate the link between cognition and fitness (Freeberg, 1996; Smith et al., 2000; White, 2010; White et al., 2010a, b, c; White, King, & West, 2002a, 2002b). No amount of spatial memory skill to find a nest can overcome an inability to find a mate and produce fertile eggs. Past work has revealed many aspects of social behavior can have overwhelming influences on the number of eggs a female may lay (White et al., 2010a, b, c). Variation in egg laying in aviaries can range from females who lay no or very few eggs up to females who lay over 40 eggs in a 2-month breeding season. If these social patterns are independent of spatial memory skills and nest prospecting, then they may be irrelevant to studying the spatial cognition – fitness connection. If they are not independent, however, it would be important to understand how cognition relates to reproductive success. While never explicitly tested up to now, examinations of cowbird breeding patterns have repeatedly led us to the conclusion that reproductive success is indeed related to cognition.

Breeding is a cognitive endeavour; females must evaluate males based on a variety of multimodal characteristics, remember them, discriminate among them, learn their behavioral proclivities, select one, and establish an effective pairbond with that one. While mate selection may involve a host of different cognitive skills, one important feature that characterizes female breeding is an attention to and regulation of space. Females seem to always be regulating spatial relationships with males and with other females (King et al., 2003a, b; Smith et al., 2000; West et al., 2002), and females often use space strategically, flying away from males and, using their chatter vocalizations, inducing males to follow them (often into trouble – getting males to engage with one another in singing bouts; Freed-Brown et al., 2006; King et al., 2003a, b; West et al., 2002). While mate selection might not be tapping into the same spatial cognition modules needed for finding nests, there may be some commonalities in the use and memory of space that are important components to both tasks. If so, spatial cognition abilities may relate to mate selection and breeding success.

For males, important requirements of reproductive success are (1) learning to sing an attractive song – one that can effectively elicit a female’s copulation solicitation display, (2) singing to females repeatedly – this is the most predictive variable associated with copulation success (White et al., 2010a, b, c), and (3) singing with other males to establish dominance (Rothstein et al., 1988). Thus, producing and singing a good song is a long-term learning problem for males and it is accomplished through singing to other males and to females and responding to the social feedback they receive (West & King, 1988). Nowicki and colleagues (Nowicki et al., 1998, 2002) have argued that because song is a challenge to learn, females (and thus evolution) may be selecting mates who are better learners. Thus, cognition and song attractiveness may be evolutionarily linked (Searcy & Nowicki, 2019; but see Templeton et al., 2014).

Song learning has always been considered a very specialized cognitive skill (Sherry & Schacter, 1987), but learning about singing and regulating the signal itself depending on the social context (Gersick & White, 2018) may be subsumed by other cognitive abilities. Males must sing to other males to establish dominance relationships. These countersinging bouts have been shown to stimulate females’ egg production (White et al., 2010a, b, c), and must be learned through interactions with other males. Juveniles raised without effective adult male tutors never learn to countersing effectively (White, King, & West, 2002a). Males must learn to regulate space effectively with other males, get close to them, temper aggressiveness, copy the behavioral patterns of their singing partner, and stay engaged for some amount of time. Males must also sing repeatedly to females. To do this effectively, they must regulate the intensity of their behavioral display depending on the female’s preferences, they must keep track of their female pairmate in space and time, and track the behavioral advances of other males. The pragmatics of singing appropriately would appear to be another cognitive challenge that may be related to learning to sing an attractive song.

### A unique fusion

The current work endeavours to examine the link between cognition and reproductive success. We take advantage of a very special set of subjects of cowbirds. These were wild-caught individuals that spent significant time in David Sherry’s cognitive behavior lab at the Advanced Facility for Avian Research (AFAR) at Western University, London, Ontario, Canada. There they engaged in an extensive series of studies than involved spatial and non-spatial cognitive tasks. At the end of their tenure at AFAR, the birds were transported to the Field Avian Research Megalab (FARM) at Wilfrid Laurier University, Ontario, Canada. At the FARM, the birds were housed in large outdoor aviaries and spent a breeding season in these aviaries with an assortment of other cowbirds. All birds had their breeding behavior and egg laying measured. In addition, males had the attractiveness of their songs measured using playback experiments. Here, for the first time, we examine the relationship between the performance of birds in the cognition lab and their breeding behavior.

Based on past work on breeding patterns, we identified a priori the breeding variables that we expected to relate to cognition. For females, we hypothesized that selecting a quality male and interacting effectively with him to create a pairbond would be related to cognition. If this is the case, then we should expect to see a relationship between cognition and reproductive success. Because cowbirds are removed from the costs associated with raising young, they can lay more eggs than parental species. If effective mate selection, coordinated courtship, and breeding behavior are related to cognition, then we expect cognition and egg production to be linked.

For males, we hypothesized that song attractiveness and singing patterns (specifically countersinging) would be related to their cognitive abilities.

## Methods

### Subjects

Twelve wild-caught adult brown headed cowbirds (six of each sex) served as subjects for this experiment. Birds were caught at Queen’s University Biological Station near Elgin Ontario Canada in 2011 and transported to AFAR. They spent 4 years there, housed individually but exposed to birds in neighboring cages in a colony room. They were provided with soft flexible and solid perches and ping pong ball toys. Birds had *ad lib* access to a modified Bronx Zoo diet for omnivorous birds – mealworms, fruits, vegetables, seed plus vitamin-treated water.

After the end of cognition testing (outlined below) in October 2015, we transported these birds to the FARM where they were housed together in a 2.5 x 2.5 x 2.5 m outdoor flight cage for 1 month before being randomly assigned into two resident flocks of wild-caught cowbirds in large outdoor aviaries. The resident birds were all adults, captured on the FARM premises, and had spent between 1 month and 1 year in the aviaries. All birds had access to *ad lib* water and food (the same modified Bronx zoo diet for omnivorous birds, plus seed, mealworms, and anything else they foraged in the outdoor aviaries). Aviaries were large 12 x 6 x 4 m outdoor facilities containing grass, trees, shrubs, perches, and an indoor shelter.

### Procedures during cognitive testing (AFAR)

The subjects plus an additional four males, and six females served in a series of delayed match to sample spatial memory and color-discrimination tasks (Guigueno et al., 2014, 2015), as well as numerical discrimination tasks (unpublished data), and were tested at two different times of the year, corresponding to breeding season and non-breeding season. Details of spatial and colour cognition testing are given in Guigueno et al. (2014, 2015). For numerical discrimination, birds were trained to locate and discriminate between two nests containing different numbers of eggs. Birds experienced 90 trials where they compared nests containing one versus four eggs, two versus three eggs, and five versus six eggs.

For examinations of actual performance on these tasks, see the published papers (Guigueno et al., 2014, 2015). Here we report only on the performance of the subjects relative to one another (within tasks) and to themselves (across tasks). To do so, we measured for each of the tasks the proportion of trials in which birds were able to complete the task correctly and then calculated each bird’s percentile ranking of their performance. We created measures of relative cognitive performance by taking the mean rankings of each bird across all of the tasks. One female who went to the FARM did not engage in any of the cognitive tests at AFAR. Her breeding data therefore could not be compared to her cognitive abilities.

### Procedures during breeding (FARM)

Total flock size for aviary 1 was 13 females and 11 males, and for aviary 2 was 14 females and 13 males. Aviaries 1 and 2 contained four and five females, respectively, who had received lesions to HVC (part of the song selectivity area of the brain) as part of a separate experiment; these females’ data are not used in this analysis. Breeding data were collected from 15 May–9 June 2016. All birds wore unique combinations of colored leg bands to permit individual identification.

#### Song censuses

Each day of the breeding season, two observers collected data in the aviaries from 6 a.m. to 10 a.m. using established protocols designed to sample singing patterns, dominance, pairbonding, and copulation success. Methodological details can be found in previous papers (White, King, & West, 2002a). Briefly, using automated speech-to-text software (White, King, & Duncan, 2002), data collectors all-occasion sampled each singing interaction, determined the identity of the singer, and the identity of the target of the song, as well as any behavioral interactions that occurred immediately following the song (copulation, fight, fly away, etc.; see Tables 1 and 2). Countersinging between males was measured automatically by programmable databases as chains of repeated singing back and forth among at least two males with no more than 15 s elapsing between songs.

**Table 1:** Means and standard errors for breeding behavior of resident and AFAR females during the breeding season at the FARM, as well as correlations between the measures from AFAR females and their spatial cognition score (R Spat) Variables include: number of chatter vocalizations recorded per female during observation sessions (* note that this correlation is based on only two AFAR females to ever chatter)

Females		Chatter	Songs rec’d	Fly away	% PM	Cops	Song att. of PM	Eggs laid
Residents	Mean	1.64	155.73	15	0.81	2.00	0.35	3.18
	SEM	0.97	20.90	2.33	0.04	0.57	0.05	0.88
AFAR	Mean	3.83	189.17	11	0.72	1.67	0.50	2.83
	SEM	2.69	44.61	3.34	0.09	0.56	0.07	1.54
	R Spat	0.968*	**0.884**	0.440	0.615	0.707	0.559	**0.905**

Songs received from males (songs rec’d), the number of times females flew away from males who were singing to them (Fly away), percent of male directed song received from the female’s pairmate (% PM; pairmate is determined by the male with whom the female copulates, or, if no copulations were recorded, the male who sings the most songs to the female), copulations (Cops), the song attractiveness (Song att.) of their pairmate (PM, as measured in playback tests), and number of eggs laid (eggs laid)

**Table 2:** Means and standard errors for breeding behavior of resident and AFAR males during the breeding season at the FARM as well as correlations between the measures from AFAR males and their spatial cognition score (R spat) and color cognition score (R col). Variables include: number of songs sung that were not directed toward any other bird per male during observation sessions (Undir)

Males		Undir	Dir M	Dir F	Cops	Fights	# PMs	Song att.	CS	Pat
Residents	Mean	28.67	291.92	212.17	3.58	4.17	1.58	0.45	100.83	5.25
	SEM	6.52	75.85	68.75	1.22	1.21	0.51	0.04	25.28	2.59
AFAR	Mean	30.67	246.17	199.67	1.67	3.67	1.33	0.45	82.50	2.67
	SEM	12.58	60.85	53.81	0.49	1.87	0.42	0.04	14.10	1.58
	R spat	0.331	0.627	-0.319	0.098	0.168	-0.062	0.559	**0.850**	0.354
	R col	0.085	0.390	0.349	0.071	0.079	-0.035	**0.901**	-0.077	0.101

Songs directed to other males (Dir M), songs directed to females (Dir F), copulations (Cops), fights with other males (Fights), number of females with whom they established a pairbond (# PMs), Song attractiveness (Song att.; as measured in playback tests), amount of countersinging sung (CS), total number of eggs sired (Paternity, Pat)

### Egg collection

Surveillance cameras positioned in trees over artificial nests recorded the identity of all females who laid eggs during the breeding season. Nests contained grass, and plaster of Paris mock eggs created from molds of cowbird eggs. Eggs were collected each morning at approximately 7 a.m.. Past work has shown, at least in aviaries, that females engage in very little extrapair reproductive activity (an analysis of 373 eggs, 160 birds in seven groups revealed all offspring were sired by pairmates; White et al., 2010a, b, c). Thus, we use the social pairbonds of the females to determine male reproductive success.

### Song testing

We assessed the attractiveness of each male’s song by recoding singing in the aviaries using shotgun Sennheiser MKH 8070 microphones recorded to a Marantz PMD670 solid state recorder and playing these songs to a set of ten unfamiliar females. Procedures for playback testing followed established protocols (King et al., 2003a, b). Briefly, for each song presentation we recorded whether the song elicited the females’ copulation solicitation display. At the end of the playback, we determined the proportion of playbacks of each song that received copulation solicitation displays from the females. The overall attractiveness score for each song was calculated by taking the mean of these proportions across all females in the playback test.

### Statistical analysis

We correlated measures of cognitive performance from AFAR to breeding season measures at the FARM. Due to the small sample sizes, we limited our analyses to those features of breeding that past work has demonstrated (a) relate to reproductive success, and (b) that we have hypothesized were most dependent on cognition.

## Results

### Cognitive performance at AFAR

Each subject tested at AFAR completed between 90 and 374 different cognitive tests. We first examined consistency in relative performance across these tests to determine where we could combine scores into single cognitive scores and where we should keep them separate. Examining all subjects tested at AFAR (not just the birds that moved to the FARM), patterns of relative consistency in performance led us to create two cognitive scores: one based on spatial cognition tasks and one on color cognition tasks because males and females demonstrated more consistency in responding within these task types than between them. For the eight females who were tested in all of the spatial and color tasks in both the breeding and non-breeding sessions, they showed an average correlation of r = .64 (p > .08) in their relative performance within retention intervals for spatial tasks in the nonbreeding session, an average correlation of r = .51 (p > .19) in performance on spatial tasks in the breeding session, and an average correlation of r = .60 (p > .11) between non-breeding and breeding sessions. Females were less consistent in performance within retention intervals in the color tasks for the nonbreeding session (average correlation, r = .32, p > .43) and for the breeding session (r = .43 p > .28), though they were consistent in their performance in color tasks between non-breeding and breeding sessions (r = .82, p > .01). Notably, however, the lowest levels of consistency among females were between color and spatial tasks (average of correlations among color and spatial tests in breeding and non-breeding sessions, r = .19 p > .65).

The seven males who were tested in all the color and spatial trials in both the breeding and the non-breeding sessions showed patterns similar to the females. They were more consistent within task type than across task type, and, also like females, they showed more consistency in spatial tasks than in colour tasks (spatial, non-breeding session average correlation: r = .65, p > .11, breeding session: r = .68, p > .09, color tasks, non-breeding session: r = .20, p > .66, breeding session: r = .32, p > .48), and they showed some consistency between breeding and non-breeding sessions for color tasks (r = .50, p > .25). Differing from females, males showed very low levels of consistency between breeding and non-breeding trials for spatial tasks (r = -0.35, p > .44). Again, there was very low consistency in performance between color and spatial tasks (average of correlations among color and spatial tests in breeding and non-breeding sessions (r = .07, p > .88)).

Taken together, while the correlations testing consistency within task type do not reach statistical significance, subjects were more consistent in their performance within tasks than across tasks. Thirteen out of 15 subjects had higher variation in their performance across trial types than within trial types (Binomial test, p = .01). These patterns of consistency and lack of consistency led us to create a spatial cognition score and a color cognition score. While negative correlation between breeding and non-breeding spatial task performance scores for males suggested that these two types of tasks should be examined separately, too few males tested at AFAR in the breeding season tests were tested at the FARM to examine this difference here. Also, too few females who came to the FARM had measures from the color tasks to permit examination of this variable. Thus, for females we restricted correlations only to spatial cognition measures.

We combined spatial and numerical discrimination tasks because the performance of the subjects who completed the number discrimination tasks were more similar to their performance in the spatial tasks (r = .56, p > .14), than to their performance in the color tasks (r = .12, p > .77). Also, there were too few subjects that completed both the spatial and the numerical discrimination tasks who moved to the FARM (four males, two females) to permit separate comparisons for these tasks. Past work on nest prospecting has suggested that these two cognitive skills (space and number discrimination) may be linked (Davies & White, 2018; White et al., 2009, 2017).

### Breeding

In the aviaries, AFAR birds bred and laid eggs at rates similar to resident birds in the flocks. AFAR females (Table 1) and males (Table 2) were not significantly different from resident females and males in any measured category. For females these variables included eggs laid, copulations, song received, percent of song received from pairmate (all independent t-tests (df = 15) < 1.06, all ps > .31). For males, variables tested included copulations, eggs sired, male-directed song sung, female-directed song sung, undirected song sung, number of pairmates, fights, countersinging, or song attractiveness (all t tests (16) < 1.07, all ps > .29)).

Taken together the lack of any differences among AFAR birds and residents suggested that AFAR birds adjusted to living in the large aviaries similar to other wild-caught cowbirds and thus their experience in the cognition lab did not influence their breeding behavior in any overt way.

### Relationships between cognition and breeding

#### Females

**Fig. 1:**
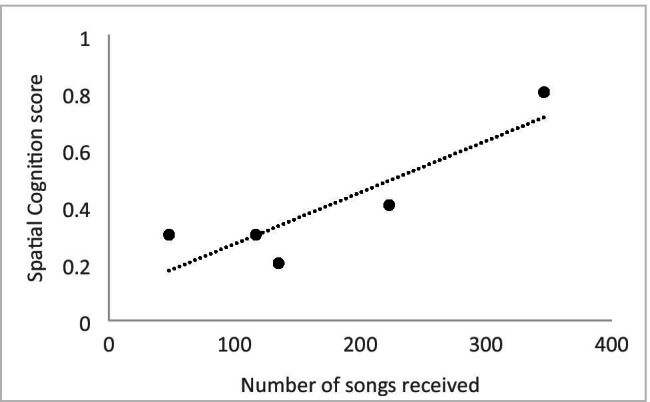
Scatterplot depicting the relationship between a females’ spatial cognition score from the Advanced Facility for Avian Research (AFAR) and the number of songs sung to her from males during the breeding season at the Field Avian Research Megalab (FARM)

**Fig. 2:**
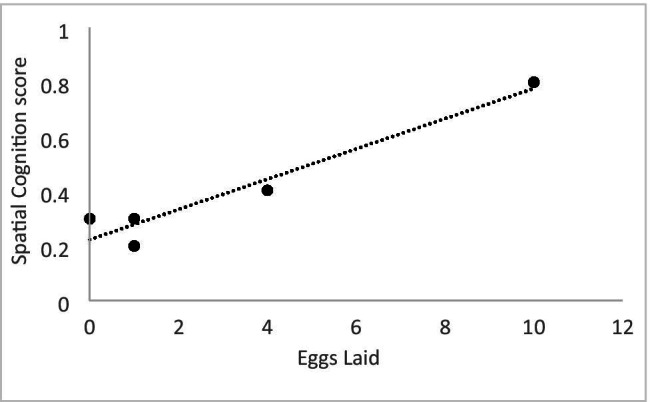
Scatterplot depicting the relationship between a female’s spatial cognition score from the Advanced Facility for Avian Research (AFAR) and the number of eggs she laid during the breeding season at the Field Avian Research Megalab (FARM)

The spatial cognition measure from AFAR studies correlated strongly with the number of songs females received from males (r = .88, N = 5, p < .05; Fig. 1). In addition, females’ spatial cognition score correlated remarkably strongly with egg production (r = .96, N = 5, p < .01; Fig. 2). Note, however, in Fig. 2 that there was one female with extremely high cognition and egg production scores.

The only other variable correlating significantly with cognition scores in females was the amount of chatter they produced (r = .97, p > .01). This relationship should be taken with caution, however, in that it is driven by only two of the females. No other AFAR females chattered. All breeding season patterns for females are provided in Table 1.

#### Males

**Fig. 3:**
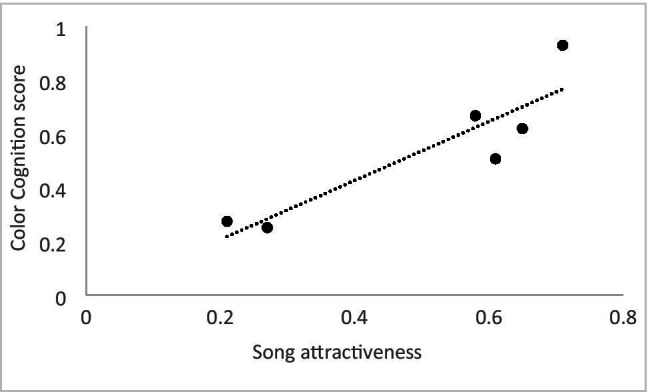
Scatterplot depicting the relationship between a male’s color cognition scores from the Advanced Facility for Avian Research (AFAR) and his song attractiveness as measured in playback tests

**Fig. 4:**
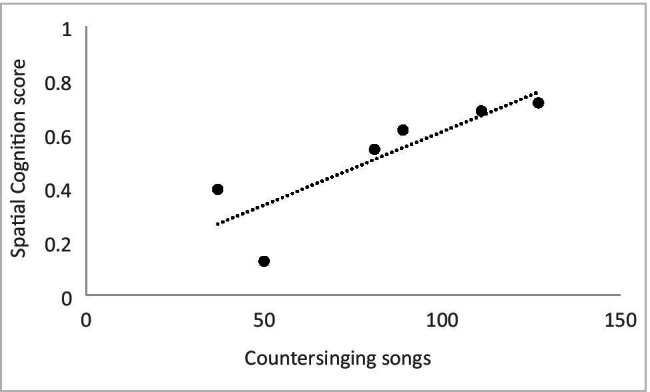
Scatterplot depicting the relationship between a male’s spatial cognition score from the Advanced Facility for Avian Research (AFAR) and the number of countersinging songs he sang during the breeding season at the Field Avian Research Megalab (FARM)

Playback results revealed a significant correlation between song attractiveness and males’ color cognition score (r = .90, N = 6, p < .02; Fig. 3). In the aviaries, color cognition did not correlate with any other breeding-related variable. Spatial memory cognition scores for males correlated with the amount of countersinging they performed (r = .85, N = 6, p < .04; Fig. 4). All breeding season patterns for males are provided in Table 2.

## Discussion

Despite the small sample size, the different cognitive scores correlated with several aspects of breeding behavior and reproductive success in both males and females.

### Females

Those females who reliably scored highest on spatial tests in the lab received the most courtship song from males. The number of songs sung to females is an important variable associated with pairbond strength and copulation success, and thus is integral to reproduction (White et al., 2010a, b, c). It is unclear what drives this correlation. It is possible that there is something about these females that makes them more attractive to males. Results of past work, however, would suggest that something about the females’ behavior is important in stimulating the males to sing to them more often (Maguire et al., 2013). What females do to get males to sing to them more often is unknown – though one possibility is the use of chatter in response to males’ song (Maguire et al., 2013).

The other variable significantly associated with cognition for females was egg production. Females are often highly variable in egg production between and across groups and past work has been only marginally successful in explaining this variation. Most of those explanations have revolved around the idea that females invest more in egg production in circumstances where they have the most valuable information about the quality of males present (White et al., 2010a, b, c). The cognition score used here is by far the strongest explanatory variable we have ever found for egg production. Perhaps females who have better cognitive abilities can best engage in the behaviors associated with selecting the highest quality, or most compatible mate, building the most successful pairbond, and therefore most likely to invest in egg production. An interesting aspect of this relationship is that laying more eggs leads to a higher spatial memory demand because it requires finding more nests. No matter what mechanism explains this relationship, the connection between spatial cognition and reproductive success suggests that sexual selection can be a driving force on spatial cognition in females.

### Males

We had the opportunity to examine how two measures of cognitive performance related to males’ fitness. First, we found that song attractiveness, as measured in playback tests related to the males’ performance on cognitive tasks that used color stimuli in delayed match-to-sample tests. That song attractiveness related to cognitive performance supports the theory that those males best able to learn are the ones who can produce the most attractive signal – a theory of the functional value of song that has been posited for songbirds in general (Nowicki et al., 1998, 2002) and cowbirds specifically (West & King, 1988). These results connecting cognitive performance and song differ from work in song sparrows (Soha et al., 2019), where no connections between cognition and song could be detected (see also Templeton et al., 2014). Why song attractiveness should relate to cognition for color per se, is unclear. Perhaps song attractiveness and performance on the color tasks are linked by another unmeasured variable relating to male quality (health, “good genes”, or stress responsiveness). This would appear unlikely since past work has shown that song attractiveness is highly dependent on developmental (West & King, 1988) and immediate (Gersick & White, 2018) social experiences. Thus, the most likely route leading to variation in song attractiveness involves interacting and learning from the visual responses of females and other males to singing overtures. The color tasks were designed as a control for spatial memory performance and not designed specifically to examine an aspect of cognition hypothesized to be important to male breeding behavior. Thus, the color tasks may be measuring some more general aspect of visual acuity, attention, or learning. More work is needed to determine exactly what cognitive mechanism is driving color discrimination and song development. It is clear, however, that the cognitive ability measured using the color task was distinct from spatial memory skill because performance on spatial tasks did not relate to song attractiveness.

Spatial task performance did, however, relate to one important aspect of singing in males: countersinging. Countersinging is a skill that males must learn in order to attain and maintain dominance among males and to stimulate the reproductive output of females (White et al., 2010a, b, c). Past work has shown that countersinging is learned by juveniles over their first year of life as they approach and sing with adult males (White, King, & West, 2002a). This ability to get close to other males, sing with them in duetting bouts, and temper aggression leads to a cascade of learning other breeding skills and is highly variable among males (White et al., 2007a, b; White, King, & West, 2002a).

No other variables for males or females reached the large effect size necessary for statistical significance (other than chatter patterns in females). There is, however, a distinction that should be made between the correlation strength needed for statistical confidence and for biological relevance. Evolution can act on very small effects. Tables 1 and 2 show some of the effects that did not reach significance but will be the subject of future work, as many of them may inform us of the potential directions of effect occurring with other variables. For example, female chatter is highly stimulating and motivating to males (Burnell & Rothstein, 1994; Freed-Brown & White, 2009; Hauber et al., 2001; Lynch et al., 2017; Snyder-Mackler & White, 2011). Perhaps the production and use of chatter is a behavioral mechanism that females use to regulate males’ behavior, stimulate courtship effort, and strengthen the pairbond (Maguire et al., 2013), leading eventually to more egg output. Other interesting positive relationships with females’ spatial cognition include the song attractiveness of their pairmate, and the amount of courtship song they receive only from their pairmate (a measure of pairbond strength we have found in the past to be important for breeding success; Maguire et al., 2013).

The disclaimers here are most likely obvious: the frustratingly low sample size highlights the challenges for neuroecology and studies of animal cognition in general where the depth of understanding of individuals’ cognitive abilities trades off against testing large numbers of subjects and therefore against generalizability and statistical power. The low number of subjects precluded more detailed statistical analyses, and we could only rely on a small number of a priori comparisons requiring very strong relationships to reject a null hypothesis. Also, the birds in this study, while wild caught, experienced years of life in abnormal contexts, raising questions about generalizability to the wild (although in the aviaries they bred in patterns very similar to the resident birds). Finally, it was not the primary goal of the cognitive experiments to subsequently study fitness. Had it been, we would have ensured that we collected measures that were more directly comparable across subjects. As is, it is not clear what cognitive modules we are examining here. The color-discrimination tasks might be measuring visual acuity, attention, learning speed, etc. Spatial cognition here also includes tasks that were focused on numerical discrimination. Thus, this work should be considered an exploratory first step that, even with the limitation inherent in these data, was surprisingly successful in demonstrating relationships between different aspects of cognition and reproductive success. This discovery will drive experiments both in the lab and in aviaries for years to come.

What do these findings mean for the adaptive specialization hypothesis about cowbird spatial memory? We still have not been able to test directly whether spatial cognition abilities allow females to successfully find and select viable nests in the wild – the critical relationship posited by the adaptive specialization hypothesis that started the work with cowbirds. With modern advances in automated tracking technology and advances in neural manipulations, this relationship may be testable in the near future. The findings reported here – that different measures of cognition related to different aspects of effective breeding – support the idea that there are functionally distinct cognitive systems as proposed by Sherry and Schacter (1987). There do seem to be different cognitive domains at work here, similar to food-caching species that show different patterns of performance depending on whether a task is spatially based or color based (Olson et al., 1995). Females’ superiority in behavioral tasks and the hippocampal size evidence suggest that the potential exists for selection to act on spatial cognition through nest-finding abilities. The interconnections between these cognitive systems and diverse aspects of breeding revealed here, however, suggests some cooption, or exaptation of the cognitive system, which significantly complicates determining how selection has acted and may act (Gould & Vrba, 1982; Sherry & Schacter, 1987). Selection may be operating on spatial memory skills for both a specialized demand on the species (finding nests), and also a non-specialized demand (selecting a mate and reproducing). This suggests there are non-additive interactions among cognitive modules and fitness.

The story of how cognition and fitness relate may not be simple, but simple stories and complex systems rarely go together. The complexity of living systems presents many different routes and strategies leading to reproductive success and thus identifying how distinct memory systems relate to fitness can be challenging. Studying the wealth of links between memory systems, however – how they can work independently and together, how they react to different environments, to past experiences and to conspecifics – and ultimately lead to organizing adaptive behavior holds the promise to fully understand the evolution of the brain and intelligence.

## Data Availability

The datasets generated during the current study are available from the corresponding author on request.
